# Case of “White Blood”

**Published:** 1850-08

**Authors:** 


*Case of "White Blood."*—Dr. Bennett mentioned, that there was at present, in the male clinical ward, a boy affected with extreme enlargement of the spleen. On examining a little of his blood microscopically, a very large amount of corpuscles was discovered, quite undistinguishable from pus corpuscles. There were many features of interest in this case; it was the second of the kind which had fallen under Dr. B.'s observation, and he proposed, at some future period, to lay before the profession the conclusions to which it led. Meanwhile, any member of the society had an opportunity of examining the case in the infirmary.—*Ibid.*

